# Self-reported attitudes versus actual practice of oxygen therapy by ICU physicians and nurses

**DOI:** 10.1186/s13613-014-0023-y

**Published:** 2014-07-25

**Authors:** Hendrik JF Helmerhorst, Marcus J Schultz, Peter HJ van der Voort, Robert J Bosman, Nicole P Juffermans, Evert de Jonge, David J van Westerloo

**Affiliations:** 1 Department of Intensive Care Medicine, Leiden University Medical Center, Leiden 2300, RC, The Netherlands; 2 Laboratory of Experimental Intensive Care and Anesthesiology, Academic Medical Center, Amsterdam 1105, AZ, The Netherlands; 3 Department of Intensive Care Medicine, Academic Medical Center, Amsterdam 1105, AZ, The Netherlands; 4 Department of Intensive Care Medicine, Onze Lieve Vrouwe Gasthuis, Amsterdam 1091, AC, The Netherlands

**Keywords:** Oxygen, Hyperoxia, Mechanical ventilation, Lung injury, Intensive care medicine, Questionnaire

## Abstract

**Background:**

High inspiratory oxygen concentrations are frequently administered in ventilated patients in the intensive care unit (ICU) but may induce lung injury and systemic toxicity. We compared beliefs and actual clinical practice regarding oxygen therapy in critically ill patients.

**Methods:**

In three large teaching hospitals in the Netherlands, ICU physicians and nurses were invited to complete a questionnaire about oxygen therapy. Furthermore, arterial blood gas (ABG) analysis data and ventilator settings were retrieved to assess actual oxygen practice in the same hospitals 1 year prior to the survey.

**Results:**

In total, 59% of the 215 respondents believed that oxygen-induced lung injury is a concern. The majority of physicians and nurses stated that minimal acceptable oxygen saturation and partial arterial oxygen pressure (PaO_2_) ranges were 85% to 95% and 7 to 10 kPa (52.5 to 75 mmHg), respectively. Analysis of 107,888 ABG results with concurrent ventilator settings, derived from 5,565 patient admissions, showed a median (interquartile range (IQR)) PaO_2_ of 11.7 kPa (9.9 to 14.3) [87.8 mmHg], median fractions of inspired oxygen (FiO_2_) of 0.4 (0.4 to 0.5), and median positive end-expiratory pressure (PEEP) of 5 (5 to 8) cm H_2_O. Of all PaO_2_ values, 73% were higher than the upper limit of the commonly self-reported acceptable range, and in 58% of these cases, neither FiO_2_ nor PEEP levels were lowered until the next ABG sample was taken.

**Conclusions:**

Most ICU clinicians acknowledge the potential adverse effects of prolonged exposure to hyperoxia and report a low tolerance for high oxygen levels. However, in actual clinical practice, a large proportion of their ICU patients was exposed to higher arterial oxygen levels than self-reported target ranges.

## Background

Oxygen supply during mechanical ventilation is a highly effective and uniformly used intervention to support oxygenation of mechanically ventilated patients in the intensive care unit (ICU). Although oxygen administration is a lifesaving strategy in the management of patients with respiratory insufficiency, the clinical implications of hyperoxia remain an important subject of debate [[1]]. The controversies are triggered by a considerable number of studies showing beneficial, harmful, and/or insignificant effects of oxygen therapy on outcome in different subgroups [[2]-[15]]. However, morbidity and mortality may be substantially impacted by the used threshold and depend on degree, duration, and susceptibility for hyperoxia.

The emerging laboratory evidence for the double-edged nature of oxygen (lifesaving but also potentially harmful) is compelling [[16]-[22]], but robust clinical studies and evidence-based guidelines in critically ill patients are still limited [[23]-[26]]. Consequently, the attitudes and beliefs in the management of oxygen administration vary considerably in clinical practice [[27],[28]]. In general, physicians are inclined to treat hypoxemia aggressively in order to achieve satisfactory tissue oxygenation [[23],[29],[30]]. However, hyperoxemia is often considered acceptable, especially when applied fractions of inspired oxygen (FiO_2_) are relatively low [[31]-[33]].

Given the lack of established guidelines on oxygen therapy in ICU patients, our study was designed to investigate the common beliefs and self-reported attitudes of ICU physicians and nurses on oxygenation targets and to compare this with actual treatment of ICU patients in the same hospitals. We hypothesized that the potential harmful effects of oxygen are well known and generally acknowledged, but that in real clinical practice, hyperoxia is not a major concern for ICU professionals.

## Methods

### Questionnaire

An anonymous online survey was performed between June and August 2012 to elicit the self-reported behavior of ICU personnel with respect to oxygen therapy. The questionnaire was a modified and comprehensive version of previously used questionnaires from Canada and Australia/New Zealand [[27],[28]] and comprised multiple choice questions (see Additional file 1). The target population consisted of physicians and nurses, working in closed format, mixed medical and surgical, tertiary care ICUs of three participating hospitals, including two academic and one large teaching hospital in the Netherlands. Participants were invited by email to complete the online questionnaire. A reminder was sent once to all participants.

### Patient data

Analyses were performed on data recorded between 1 April 2011 and 31 March 2012 for all patients admitted to the ICU departments of the same hospitals that participated in the questionnaire study. Anonymous encrypted data were collected from the patient data management system (PDMS) database (MetaVision, iMDsoft, Leiden, The Netherlands). According to the Dutch Medical Research Involving Human Subjects Act, there was no need for informed patient consent or approval by ethical committees, as only registries without patient identifying information were used.

**Figure 1:**
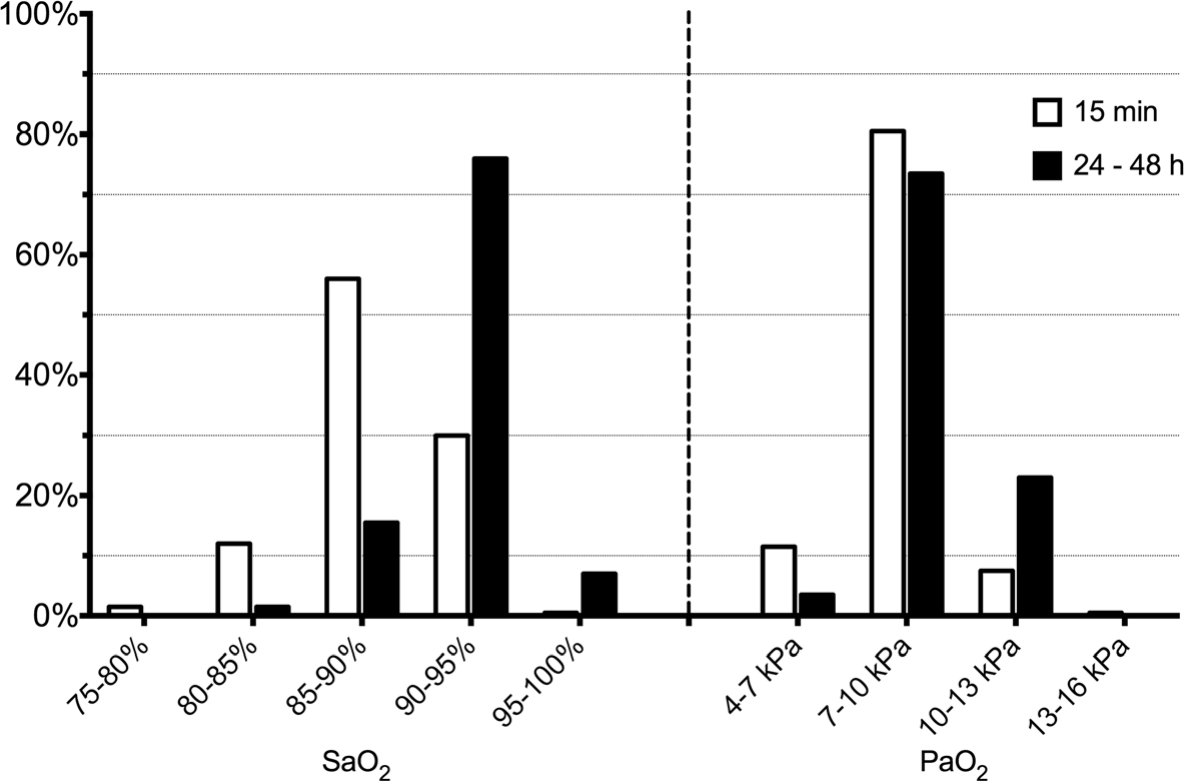
**Self-reported tolerance limits for short-term (15 min, open bars) and longer term (24 to 48 h, closed bars) oxygenation.** Bars represent percentage of respondents (*n* = 200). The presented case is a young to middle-aged ARDS patient in the ICU requiring mechanical ventilation. Ventilator settings (e.g., PEEP, airway pressures, I/E ratio, flow ratio) are optimized with respect to the PaO_2_/FiO_2_ ratio and hemodynamic indices. Lung injury due to high FiO_2_ and/or ventilator settings is minimized. There is no evidence to indicate end-organ ischemia, and hemodynamics are stable.

Arterial blood gas (ABG) analyses and concurrent ventilator settings were extracted to retrospectively assess actual practices regarding oxygen therapy. Data from ICU admission to dismissal or death were included for analysis. Data with a partial arterial oxygen pressure (PaO_2_) of ≤4.5 kPa or 33.8 mmHg (*n* = 209) were excluded to prevent confounding by venous blood gas samples. Further exclusion of samples with PaO_2_ of 4.5 to 5.0 kPa (*n* = 146) or PaO_2_ of 5.0 to 6.0 kPa (*n* = 356) did not materially change our observations (data not shown). Every set of ABG data and ventilator settings was compared with the following set, as described previously [[32]]. Prone positioning, recruitment maneuvers, and other efforts that may improve oxygenation could not be explored in this database. Clinicians' responses to ABG results were explored by analyzing the FiO_2_ and positive end-expiratory pressure (PEEP) adjustments in a subsample of mechanically ventilated patients when more than two ABG samples and ventilator settings were recorded. PaO_2_ values were categorized according to the commonly self-reported acceptable range (7 to 10 kPa or 52.5 to 75 mmHg) extracted from the survey results (Figure 1). This range is roughly consistent with oxygenation goals (7.3 to 10.7 kPa or 55 to 80 mmHg) previously suggested by the Acute Respiratory Distress Syndrome (ARDS) network [[30],[34]].

### Mechanical ventilation protocol

Local guidelines, applicable during the survey and collection of ABG and ventilation data, instructed for lowest acceptable FiO_2_ levels. FiO_2_ levels higher than 60% should be avoided by increasing PEEP levels, instituting inverse-ratio ventilation or prone positioning. No explicit target ranges for PaO_2_, FiO_2_, or saturation were specified in the participating hospitals during this period.

### Statistical analysis

All data are expressed as percentages of the total number of respondents for the particular questions, unless otherwise specified. Data are presented as means (±SD) or medians (interquartile range (IQR)) depending on data distributions, unless stated otherwise. For assessing differences between physicians and nurses, ICU personnel were grouped according to their respective profession and data were analyzed using Fisher's exact tests. ICU physicians, fellows, and residents were classified as physicians; ICU nurses and ICU nurses in training were classified as nurses.

Statistical analyses were conducted using STATA/SE 10.1 (StataCorp LP, College Station, TX, USA).

## Results

### Data derived from questionnaire

#### Respondent characteristics

Approximately 500 potential participants were invited to complete the online questionnaire. Full or partial responses were received from 215 ICU physicians and nurses with a mean age of 40.4 ± 10.0 years (range 24 to 62). In total, 171 (80%) respondents fully completed all questions. The group of respondents consisted of 28 (13%) critical care physicians, 15 (7%) fellows, 15 (7%) residents, 141 (66%) ICU nurses, 11 (5%) ICU nurses in training, and 5 (2%) ICU clinicians with another type of practice.

#### Opinions towards oxygen toxicity

The responses are listed in Table 1. Overall, 126 (59%) respondents considered oxygen-induced lung injury in mechanically ventilated patients a major concern. However, the vast majority of respondents (81%) considered high tidal volumes and high inspiratory pressures as the greatest risk for lung injury in mechanically ventilated patients. No differences between physicians and nurses were detected.

**Table 1:** Questionnaire responses regarding risks assessment and management in oxygen therapy

**Question**	**Responses (% of total)**	**Physicians vs. nurses**
Is oxygen induced lung injury a concern when placing a patient on mechanical ventilation?		NS
YES, a major concern	126 (59%)	
due to the *high incidence* of injury	13 (6%)	
due to the *severity* of injury	63 (29%)	
due to the *high incidence and severity* of injury	50 (23%)	
YES, but not a *major* concern	80 (37%)	
NO, it is not a concern	9 (4%)	
In your opinion, which one of the following two situations poses a greater threat of lung injury for mechanically ventilated patients?		NS
High FiO_2_	35 (16%)	
High tidal volumes and high ventilator pressures	173 (81%)	
Don't know	7 (3%)	
In situations when maximal SaO_2_ achievable is low (±85%) or when FiO_2_ requirements are high, do you assess indices of tissue oxygenation?		*P* = 0.05
NO	91 (43%)	
YES, lactate	88 (42%)	
YES, microcirculation with OPS/SDF imaging	4 (2%)	
YES, a combination of indices	20 (9%)	
YES, SvO_2_	6 (3%)	
YES, other	2 (1%)	

NS, not significant; FiO_2_, fractions of inspired oxygen; OPS, orthogonal polarization spectral; SDF, sidestream dark field; SaO_2_, arterial oxygen saturation; SvO_2_, mixed venous oxygen saturation.

#### Self-reported acceptance of hyperoxemia and hypoxemia

The percentages of respondents accepting various oxygenation ranges in a young to middle-aged mechanically ventilated patient with ARDS are shown in Figure 1.

For both short and longer lasting periods, the vast majority of respondents choose 7 to 10 kPa (52.5 to 75 mmHg) as the lowest acceptable PaO_2_ range. Physicians were more tolerant towards lower PaO_2_ limits for short duration than nurses (*P* < 0.001).

Presented with a patient whose arterial oxygen saturation (SaO_2_) levels are low (<85%) or FiO_2_ requirements are high, indices of tissue oxygenation were frequently assessed (Table 1). Differences between physicians and nurses approached statistical significance (*P* = 0.05), with physicians favoring lactate assessment and nurses being less likely to demand some assessment of tissue oxygenation. Nurses in training more often favored lactate assessment than senior ICU nurses (*P* = 0.01).

#### Adjustment of FiO_2_ in specific clinical cases

The proportions of ICU clinicians adjusting FiO_2_ levels in specified clinical cases are listed in Table 2. Observed differences by profession were mainly restricted to questions regarding patients with untreatable anemia, where physicians generally favored higher FiO_2_ levels than nurses. Only minor differences within the clustered categories of physicians (comparison between physicians, fellows, residents) and nurses (ICU nurses, nurses in training) were observed.

**Table 2:** **Percentages of respondents adjusting FiO**_**2**_**levels in specified clinical conditions with presented levels of arterial oxygenation**

	**ARDS**			**Cardiac ischemia**			**Cerebral ischemia**			**Sepsis**			**Untreatable anemia**		
	**FiO**_**2**_**response**			**FiO**_**2**_**response**			**FiO**_**2**_**response**			**FiO**_**2**_**response**			**FiO**_**2**_**response**		
	**Higher**	**Unchanged**	**Lower**	**Higher**	**Unchanged**	**Lower**	**Higher**	**Unchanged**	**Lower**	**Higher**	**Unchanged**	**Lower**	**Higher**	**Unchanged**	**Lower**
SaO_2_															
80% to 85%	97.4	2.6	0.0	100	0.0	0.0	98.9	1.1	0.0	99.5	0.5	0.0	92.4	7.1	0.5
85% to 90%	61.5	38.5	0.0	96.4	3.6	0.0	91.4	8.0	0.5	92.5	7.0	0.5	85.3	13.6	1.1
90% to 95%	4.1	78.5	17.4	42.6	54.9	2.6	23.0	71.7	5.3	19.3	75.9	4.8	57.6	36.4	6.0
95% to 100%	0.0	17.4	82.6	2.6	61.0	36.4	0.5	41.2	58.3	0.5	40.1	59.4	13.6	58.7	27.7
PaO_2_															
6 kPa	96.6	3.4	0.0	98.3	1.7	0.0	98.3	1.7	0.0	98.3	1.7	0.0	96.5	3.5	0.0
9 kPa	9.0	85.3	5.6	60.1	38.2	1.7	40.6	58.9	0.6	37.7	60.6	1.7	66.7	31.6	1.8
12 kPa	0.6	27.1	72.3	5.6	60.7	33.7	2.3	53.7	44.0	2.9	47.4	49.7	22.2	52.6	25.1
16 kPa	0.0	2.3	97.7	2.2	10.1	87.6	1.1	4.6	94.3	0.6	7.4	92.0	6.4	25.7	67.8

All clinical situations represent patients in the ICU, who have been invasively mechanically ventilated for at least 5 days, with FiO_2_ set at 50%. ARDS = patient with acute respiratory distress syndrome and pneumonia; cardiac ischemia = patient with signs of cardiac ischemia (ST-depressions in the anterior leads [max 3 mm]) and pneumonia; cerebral ischemia = patient with recent cerebral ischemia and one-sided hemiplegia; sepsis = patient with a liver abscess and sepsis; untreatable anemia = Jehovah's Witness with stable hemoglobin of 1.8 mmol/L after gastric bleeding; higher = increase FiO_2_, higher than current 50%; unchanged = maintain FiO_2_ at current 50%; lower = decrease FiO_2_, lower than current 50%.

### Data derived from ABG measurements and ventilator settings

#### Descriptive data

A total of 107,888 ABG results with concurrent ventilator settings, covering 5,565 patient admissions, were retrieved and included for analysis over a 1-year period prior to the survey in three hospitals. Median interval between two consecutive ABG samples was 214 min (IQR 130 to 331), and the median number of ABG samples per patient was 7 (IQR 4 to 19). Mean PaO_2_ was 12.9 kPa (SD 5.1) or 96.8 mmHg, and median PaO_2_ was 11.7 kPa (IQR 9.9 to 14.3) or 87.8 mmHg. Overall, in 25.3% of ABG results, PaO_2_ was in the self-reported range (7 to 10 kPa), 1.2% was lower and 73.4% was higher than the predefined range.

Mechanical ventilation settings showed a mean PEEP of 6.1 cm H_2_O (SD 4.3), and median PEEP was 5 (IQR 5 to 8). Mean FiO_2_ was 0.45 (SD 0.14), and median FiO_2_ was 0.40 (IQR 0.40 to 0.50). Only small differences were observed between the three participating hospitals.

#### Recorded FiO_2_ adjustments following ABG analysis

After exclusion of spontaneously breathing and non-invasively ventilated patients, 62,875 ABG records derived from 4,264 mechanically ventilated patients were included for analysis of FiO_2_ adjustments in response to ABG samples.

Analyzing every first registered ABG sample in the ICU, 62,222 PaO_2_ measurements covering 3,791 patients were retrospectively categorized in predefined ranges and were followed by a recorded FiO_2_ adjustment. The subsequently registered PaO_2_ measurement was compared with the first registered PaO_2_ (Table 3).

**Table 3:** **FiO**_**2**_**adjustment following ABG analysis and its effects on oxygenation measured in the next ABG**

**PaO**_**2**_**(%)**	**Adjustment of FiO**_**2**_**(%)**	**Successive PaO**_**2**_		
		**[*n*** **= 61,073]**		
**[*n*** **= 107,888]**	**[*n*** **= 62,222]**	**Higher**	**Unchanged**	**Lower**
		**(Delta PaO**_**2**_**)**		**(Delta PaO**_**2**_**)**
<7 kPa (1.2)	Higher (34.7)	96.6% (+5.3)	0.4%	3.0% (−0.5)
	Unchanged (46.9)	87.4% (+5.6)	3.2%	9.4% (−0.6)
	Lower (18.4)	95.1% (+7.9)	2.4%	2.5% (−0.9)
7 to 10 kPa (25.3)	Higher (27.9)	76.3% (+3.1)	2.7%	21.0% (−0.9)
	Unchanged (56.0)	66.3% (+2.0)	4.3%	29.4% (−0.8)
	Lower (16.1)	61.3% (+2.6)	3.6%	35.1% (−1.0)
>10 kPa (73.4)	Higher (10.8)	48.6% (+4.6)	2.1%	49.3% (−3.1)
	Unchanged (62.0)	44.7% (+2.1)	3.1%	52.2% (−2.3)
	Lower (27.2)	23.5% (+2.4)	1.7%	74.8% (−4.6)
Total (100)	-	46.3% (+2.6)	2.7%	51.0% (−2.9)

Data presented as percentages of total and irrespective of adjustment of other ventilator settings (e.g., PEEP, I/E ratio). PaO_2_ = any recorded PaO_2_ stratified by self-reported ranges; successive PaO_2_ = PaO_2_ from successively registered ABG sample; Delta = mean difference between two successive ABG samples; Higher = increased FiO_2_ (column 2) or PaO_2_ (column 3), higher than previous level; unchanged = FiO_2_ or PaO_2_ equal to previous level; lower = decreased FiO_2_ or PaO_2_, lower than previous level. A total of 62,222 PaO_2_ measurements from 3,791 patients (57.7% of all 107,888 ABG samples) in the database is followed by an adjustment of ventilator settings, and 98.2% of PO_2_ measurements is followed by a successive PO_2_ measurement (*n* = 61,073) in the same patient when adjustment of FiO_2_ is also measured.

Table 4 shows that quantity, direction, and magnitude of ventilator adjustments in response to high arterial oxygen levels are considerably influenced by the level of FiO_2_. In 58.3% of cases with PaO_2_ higher than the upper level of the commonly self-reported acceptable oxygenation (10 kPa or 75 mmHg), neither FiO_2_ nor PEEP levels had been lowered when the next ABG sample was taken.

**Table 4:** Adjustment of mechanical ventilation settings following ABG analysis

	**FiO**_**2**_**25% to 40% (*n* = 37,172)**			**FiO**_**2**_**40% to 60% (*n* = 23,466)**			**FiO**_**2**_**60% to 80% (*n* = 4,318)**			**FiO**_**2**_** 80% to 100% (*n* = 2,041)**			**Total (*n* = 68,222)**		
PaO_2_	13.4 (4.1)			12.6 (5.2)			12.3 (6.6)			16.0 (13.7)			13.1 (5.2)		
SpO_2_	98.0 (2.9)			96.5 (3.8)			94.3 (6.3)			89.7 (12.5)			96.9 (4.6)		
PEEP	5.6 (3.4)			7.3 (4.4)			9.1 (5.6)			9.0 (6.3)			6.5 (4.2)		
**PaO**_**2**_**range (kPa)**	**Delta FiO**_**2**_**(%)**	**Delta PEEP (cm H**_**2**_**O)**	**No decrease in FiO**_**2**_**or PEEP (%)**	**Delta FiO**_**2**_**(%)**	**Delta PEEP (cm H**_**2**_**O)**	**No decrease in FiO**_**2**_**or PEEP (%)**	**Delta FiO**_**2**_**(%)**	**Delta PEEP (cm H**_**2**_**O)**	**No decrease in FiO**_**2**_**or PEEP (%)**	**Delta FiO**_**2**_**(%)**	**Delta PEEP (cm H**_**2**_**O)**	**No decrease in FiO**_**2**_**or PEEP (%)**	**Delta FiO**_**2**_**(%)**	**Delta PEEP (cm H**_**2**_**O)**	**No decrease in FiO**_**2**_**or PEEP (%)**
<7	+6.1 (13.8)	+0.3 (3.2)	87.6	+5.4 (13.2)	+0.7 (3.7)	81.3	+2.0 (14.5)	+0.6 (6.0)	86.3	−7.0 (16.8)	+1.0 (6.7)	76.9	+3.0 (14.8)	+0.7 (4.3)	83.3
7 to 10	+2.6 (6.7)	+0.1 (3.1)	81.9	+1.6 (7.3)	+0.2 (4.0)	80.3	−0.2 (9.4)	+0.3 (5.2)	80.8	−6.7 (14.8)	+0.1 (5.9)	76.5	+1.3 (8.3)	+0.2 (3.8)	80.8
10 to 15	+0.6 (4.9)	−0.2 (2.7)	72.9	−1.5 (6.6)	−0.2 (3.8)	48.2	−5.2 (9.2)	+0.1 (4.9)	33.7	−15.6 (19.2)	+0.4 (5.7)	31.1	−0.6 (6.7)	−0.1 (3.2)	62.3
15 to 20	−0.3 (5.1)	−0.3 (2.7)	65.0	−4.8 (7.2)	−0.3 (3.3)	25.1	−11.2 (11.4)	+0.1 (4.7)	13.5	−20.4 (20.5)	+0.0 (4.7)	23.6	−2.0 (7.3)	−0.2 (3.0)	52.8
20 to 25	−1.2 (6.4)	−0.2 (2.4)	54.2	−6.1 (9.0)	−0.4 (3.0)	22.1	−12.7 (12.4)	−0.4 (5.3)	11.8	−25.9 (21.7)	−0.1 (4.8)	21.3	−4.2 (10.1)	−0.3 (2.9)	40.4
25 to 30	−1.6 (7.0)	−0.4 (2.8)	51.6	−7.2 (9.6)	−0.3 (2.7)	18.8	−19.0 (12.1)	−0.4 (3.8)	9.2	−25.3 (21.6)	−0.1 (4.8)	22.9	−7.1 (12.2)	−0.3 (2.9)	32.0
>30	−1.0 (6.6)	−0.4 (4.0)	69.8	−3.5 (9.6)	−0.4 (4.5)	43.0	−15.4 (15.8)	+0.3 (5.7)	24.8	−33.6 (23.2)	−0.4 (4.0)	14.3	−5.7 (15.9)	−0.3 (4.2)	51.6

Data are means (±SD), unless stated otherwise. Delta = difference between two successive ABG samples.

## Discussion

In accordance with accumulating laboratory evidence for the toxic effects of oxygen in pulmonary injury [[1],[35]-[38]], the majority of surveyed ICU physicians and nurses consider prolonged hyperoxic exposure to be associated with an increased risk for lung injury, although a lower risk than high tidal volumes and inspiratory pressures. In contrast, in actual clinical practice, the majority of PaO_2_ values recorded in ICU patients are higher than recommended targets under comparable conditions and are generally accepted by ICU physicians and nurses without adjustment of ventilator settings.

Compared with previous studies from other countries, more respondents identified oxygen toxicity as a major threat to lung injury in ICU patients (59% compared with 26% and 51% in studies from Australia and Canada, respectively) [[27],[28]]. Similar proportions of respondents considered high inspiratory oxygen concentrations a more important risk than high tidal volumes or inspiratory pressures, and a similar heterogeneity in self-reported attitudes regarding oxygen therapy was observed [[27],[28],[39]]. The current results show generally higher minimum allowable SaO_2_ ranges than data from Canadian intensivists [[28]], which may indicate that clinicians' beliefs have changed over the last decade or it may merely reflect geographical differences in oxygen therapy.

It appears that clinicians' opinions regarding optimal oxygen therapy are more variable in case SaO_2_ is presented compared to PaO_2_ as marker of oxygenation. For PaO_2_, the vast majority of clinicians choose 7 to 10 kPa (52.5 to 75 mmHg), whereas the preferred targets for saturation varied between 85% and 95%. Assuming that oxygenation targets should be in line with the best evidence in available guidelines, these preferred ranges may be triggered by recommendations and protocols providing comparable PaO_2_ targets [[30],[40]]. However, caution is urged when interpreting pulse oximetry to differentiate between hyperoxemia and normoxia. Saturation levels above 95% require special attention, since the corresponding PaO_2_ levels usually cover a wide range and may substantially exceed target levels [[24],[41]].

According to the results from our questionnaire, the vast majority of respondents stated they would lower FiO_2_ levels if PaO_2_ was higher than 12 kPa (90 mmHg) or SaO_2_ was higher than 95% in ARDS patients. The proportion of respondents that would lower FiO_2_ is much lower if patients were presented with sepsis, cardiac and cerebral ischemia, or untreatable anemia. Unfortunately, we do not know whether respondents believe that oxygen is specifically harmful in patients with pre-existing acute lung injury or that higher oxygen levels are considered desirable in patients with ischemia or anemia. The latter hypothesis appears plausible, even though hyperoxemia has been reported to induce important vasoconstriction, which may lead to a paradoxical decrease in oxygen delivery [[4],[42]].

The self-reported low tolerance for higher PaO_2_ or SaO_2_ than target levels appears to be in contrast with actual treatment of patients in the same three ICUs where the survey was conducted. Neither FiO_2_ nor PEEP was changed in the majority of cases when PaO_2_ was higher than 15 kPa (112.5 mmHg) and FiO_2_ was 40% or lower. In cases when FiO_2_ was 40% to 100%, ventilator settings were adjusted more often, but even in these circumstances, hyperoxemia was accepted in approximately 20%. Considering the absence of definitive guidelines and robust controlled clinical evidence, this behavior in itself may still be justifiable [[43]]. However, the contrast between self-reported attitudes towards oxygen therapy on the one hand and actual treatment by the same healthcare workers on the other hand is striking.

The findings about oxygenation in ICU patients are consistent with previous findings from single center studies, showing that hyperoxemia was frequently present in mechanically ventilated patients and seldom led to adjustment of ventilator settings [[31],[32]]. Clinicians may have specific reasons not to adjust ventilator settings when PaO_2_ levels are higher than the target range. Indeed, we identified a considerable number of cases in which a presumed inadequate adjustment ultimately proved reasonable in the subsequent ABG sample (e.g., high PaO_2_ followed by an increase in FiO_2_, but resulting in a lower PaO_2_). These cases may reflect scenarios in which clinicians anticipate deterioration in oxygenation or otherwise consider PaO_2_ values as erroneous (e.g., arguably high PaO_2_ - see Table 4, row PaO_2_ > 30) or not representative for the current situation of a patient. Alternatively, it may be argued that hypoxemia harbors greater inherent hazards than hyperoxemia [[3]].

The differences between self-reported attitudes and actual treatment of patients should be interpreted with caution. First, the cases presented to the respondents included only limited details and do not reflect the complexity of clinical situations in daily practice. Further, we presented SaO_2_ and PaO_2_ categorized in ranges that were arbitrarily chosen. This may have influenced interpretation of the hypothetical cases. Second, ICU clinicians may have given more favorable responses in the online questionnaire due to social desirability and attention bias, although this is less likely as anonymous evaluation was secured. Third, the respondents were asked for the minimum allowable range in a specific ARDS case vignette, which may not reflect their beliefs regarding oxygen therapy in general. In the analysis of actually achieved oxygenation, we studied *all* patients independent of admission diagnosis. Also, response rates for the survey were relatively modest. However, the profession distribution in the group of respondents closely reflects a typical staff constitution in a general ICU in the Netherlands, which reduces the chance of sampling bias. In the Dutch clinical setting where respiratory therapists are not available, it is often the bedside nurse that responds first to changes in oxygenation. Therefore, the opinions of ICU nurses about oxygen therapy are important in the actual care of critically ill patients [[39]]. Finally, some ABG samples, taken shortly after ICU arrival, may reflect oxygen therapy initiated on the operating room and influenced by anesthesiological ventilation strategies. However, successive ventilator adjustments were all recorded on the ICU and were supervised by critical care physicians. Therefore, high PaO_2_ values in the direct postoperative period are not a plausible explanation for the low proportion of hyperoxic ABG samples not followed by adaptation of the ventilator settings.

Strengths of this study include the large sample of questionnaire responses and the extensive set of ABG data, derived from the same ICUs where the questionnaire was conducted. This facilitated a comprehensive comparison between self-reported attitudes and actual practices of oxygen therapy for both physicians and nurses. Further, the design of the present questionnaire closely resembles previous surveys from Canada and Australia, thereby exploring geographical patterns and trends in time concerning oxygen therapy. Our study extends these data as we have assessed objective data in our analysis including the successively measured PaO_2_ after FiO_2_ adjustment. This allows further estimation of the effects of recorded FiO_2_ adjustments in comparison with previous data [[32]].

## Conclusions

This study shows that most clinicians acknowledge the potential adverse effects of prolonged exposure to hyperoxia, in accordance with emerging evidence for pulmonary toxicity and increased risk of poor outcome in both humans and animals caused by excessive oxygenation [[2],[4],[6],[8],[16],[18],[20],[35],[44]]. However, objective data also suggest that clinicians did not consistently accommodate this conception in actual clinical practice and a large proportion of patients was exposed to arterial oxygen levels higher than self-reported as acceptable by nurses and physicians. Additional education, feedback, and implementation strategies, aimed at careful titration of oxygen, may therefore be an effective approach for strict adherence to oxygenation targets [[45]]. Studies on the effects of different target ranges for PaO_2_ on clinically relevant endpoints are needed to guide ICU professionals on how much oxygen should be administered to their patients.

## Abbreviations

ABG: arterial blood gas

FiO_2_: fractions of inspired oxygen

ICU: intensive care unit

PaO_2_: partial arterial oxygen pressure

PDMS: patient data management system

PEEP: positive end-expiratory pressure

SaO_2_: arterial oxygen saturation

## Competing interests

The authors declare that they have no competing interests.

## Authors’ contributions

HJFH drafted the manuscript and participated in the conception and design, and collection, analysis, and interpretation of the data. MJS and PHvdV participated in the conception and design, interpretation of data, and critical revision of the article for important intellectual content. RJB was involved in the collection, assembly and interpretation of data, and critical revision. NPJ participated in the interpretation of data and critical revision. EdJ and DJvW were involved in the conception and design, interpretation and collection of data, and critical revision. All authors read and approved the final manuscript.

## Additional file

## Supplementary Material

Additional file 1 **Questionnaire (translated from Dutch) as sent to all participants.** The questionnaire is a modified and comprehensive version of previously used questionnaires [[27],[28]]. Click here for file
