# Supplementary material for: Self-reported attitudes versus actual practice of oxygen therapy by ICU physicians and nurses
Source: Ann Intensive Care. 2014 Jul 25;4:23. doi: 10.1186/s13613-014-0023-y (PMC4240734; doi:10.1186/s13613-014-0023-y)
Supplement: Additional file 1 — Questionnaire (translated from Dutch) as sent to all participants. The questionnaire is a modified and comprehensive version of previously used questionnaires [[27],[28]]. [file s13613-014-0023-y-S1.pdf]

Appendix

Questionnaire (translated)
Modified and comprehensive version of previously used questionnaires [18, 19]

Q-1 In which hospital do you work?
- Academic Medical Center
- Leiden University Medical Center
- Onze Lieve Vrouwe Gasthuis

Q-2 What is your function in the hospital?
- Intensivist
- ICU fellow
- Resident
- ICU nurse
- ICU nurse in training
- Other (please specify)

Q-3 What is your age?
--

Q-4 Is oxygen induced lung injury a concern when placing a patient on mechanical ventilation?
- YES, a major concern due to the *high incidence* of injury
- YES, a major concern due to the *severity* of injury
- YES, a major concern due to the *high incidence and severity* of injury
- YES, but not a *major* concern
- NO, it is not a concern

Q-5 In your opinion, which one of the following two situations poses a greater threat of lung injury for mechanically ventilated patients?
- High FiO_2
- High tidal volumes and high ventilator pressures
- Don't know

Q-6 In situations when maximal SaO_2 achievable is low, say 85%, or when FiO_2 requirements are high, do you assess indices of *tissue* oxygenation?
- NO
- YES, lactate
- YES, microcirculation with OPS/SDF imaging
- YES, but a different index or a combination (see other)
- YES, other (please specify)

Q-7 to Q-11 pertain to young and middle-aged ARDS patients in the ICU requiring mechanical ventilation. Please assume that all ventilator settings (PEEP, airway pressure, I:E ratio, flow rates, with minimal auto-PEEP) are optimized with respect to the PaO_2/FiO_2 ratio and hemodynamic indices. Lung injury due to high FiO_2 and/or ventilator settings is minimized. There is no evidence to indicate end-organ ischemia, and hemodynamics are stable

Q-7 Independent of FiO_2, what is the minimum level of arterial oxygen saturation (SaO_2) allowable for a duration under 15 minutes?

95-100%
90-95%
85-90%
80-85%
75-80%

Q-8 Independent of FiO_2, what is the minimum level of arterial oxygen tension (PaO_2) allowable for a duration under 15 minutes?

4-7 kPa
7-10 kPa
10-13 kPa
13-16 kPa

Q-9 Independent of FiO_2, what is the minimum level of arterial oxygen saturation (SaO_2) allowable when the duration is between 24-48 hours?

95-100%
90-95%
85-90%
80-85%
75-80%

Q-10 Independent of FiO_2, what is the minimum level of arterial oxygen tension (PaO_2) allowable when the duration is between 24-48 hours?

4-7 kPa
7-10 kPa
10-13 kPa
13-16 kPa

Q-11 Independent of FiO_2, after what duration would a stable SaO_2 of 85% begin to raise concerns?

< 2 hours
2 – 24 hours
24 – 48 hours
48 – 72 hours
> 72 hours

--

Q-12 to Q-31 pertain to a situation when a patient must be placed on mechanical ventilation for at least 5 days, and FiO_2 = 50% please indicate whether you would increase, decrease or not change the level of FiO_2 for each corresponding level of arterial saturation or arterial oxygen tension.

Q-12 Patient admitted to the ICU with pneumonia and ARDS.

a. SaO_2 = 80-85% b. 6 kPa

a.	b.
Increase	Increase
Decrease	Decrease
Not change	Not change

Q-13 Patient admitted to the ICU with pneumonia and ARDS.

a. SaO_2 = 85-90% b. 9 kPa

Increase Increase
Decrease Decrease
Not change Not change

Q-14 Patient admitted to the ICU with pneumonia and ARDS.

a. SaO_2 = 90-95% b. 12 kPa

Increase Increase
Decrease Decrease
Not change Not change

Q-15 Patient admitted to the ICU with pneumonia and ARDS.

a. SaO_2 = 95-100% b. 16 kPa

Increase Increase
Decrease Decrease
Not change Not change

Q-16 Patient admitted to the ICU with signs of cardiac ischemia (ST-depressions in the anterior leads [max 3mm]) and pneumonia.

a. SaO_2 = 80-85% b. 6 kPa

Increase Increase
Decrease Decrease
Not change Not change

Q-17 Patient admitted to the ICU with signs of cardiac ischemia (ST-depressions in the anterior leads [max 3mm]) and pneumonia.

a. SaO_2 = 85-90% b. 9 kPa

Increase Increase
Decrease Decrease
Not change Not change

Q-18 Patient admitted to the ICU with signs of cardiac ischemia (ST-depressions in the anterior leads [max 3mm]) and pneumonia.

a. SaO_2 = 90-95% b. 12 kPa

Increase Increase
Decrease Decrease
Not change Not change

Q-19 Patient admitted to the ICU with signs of cardiac ischemia (ST-depressions in the anterior leads [max 3mm]) and pneumonia.

b. SaO_2 = 95-100% b. 16 kPa

Increase Increase
Decrease Decrease
Not change Not change

Q-20 Patient admitted to the ICU with recent cerebral ischemia and one-sided hemiplegia.

a. SaO_2 = 80-85% b. 6 kPa

Increase Increase
Decrease Decrease
Not change Not change

Q-21 Patient admitted to the ICU with recent cerebral ischemia and one-sided hemiplegia.

a. SaO_2 = 85-90% b. 9 kPa

Increase Increase
Decrease Decrease
Not change Not change

Q-22 Patient admitted to the ICU with recent cerebral ischemia and one-sided hemiplegia.

a. SaO_2 = 90-95% b. 12 kPa

Increase Increase
Decrease Decrease
Not change Not change

Q-23 Patient admitted to the ICU with recent cerebral ischemia and one-sided hemiplegia.

c. SaO_2 = 95-100% b. 16 kPa

Increase Increase
Decrease Decrease
Not change Not change

Q-24 Patient admitted to the ICU with liver abcess and sepsis.

a. SaO_2 = 80-85% b. 6 kPa

Increase Increase
Decrease Decrease
Not change Not change

Q-25 Patient admitted to the ICU with liver abcess and sepsis.

a. SaO_2 = 85-90% b. 9 kPa

Increase Increase
Decrease Decrease
Not change Not change

Q-26 Patient admitted to the ICU with liver abcess and sepsis.

b. SaO_2 = 90-95% b. 12 kPa

Increase Increase
Decrease Decrease

Not change Not change

Q-27 Patient admitted to the ICU with liver abcess and sepsis. SaO_2 = 95-100%

d. SaO_2 = 95-100% b. 16 kPa

Increase Increase
Decrease Decrease
Not change Not change

Q-28 Jehovah's Witness admitted to the ICU with stable hemoglobin of 1.8 mmol/L after gastric bleeding.

a. SaO_2 = 80-85% b. 6 kPa

Increase Increase
Decrease Decrease
Not change Not change

Q-29 Jehovah's Witness admitted to the ICU with stable hemoglobin of 1.8 mmol/L after gastric bleeding.

a. SaO_2 = 85-90% b. 9 kPa

Increase Increase
Decrease Decrease
Not change Not change

Q-30 Jehovah's Witness admitted to the ICU with stable hemoglobin of 1.8 mmol/L after gastric bleeding.

a. SaO_2 = 90-95% b. 12 kPa

Increase Increase
Decrease Decrease
Not change Not change

Q-31 Jehovah's Witness admitted to the ICU with stable hemoglobin of 1.8 mmol/L after gastric bleeding.

a. SaO_2 = 95-100% b. 16 kPa

Increase Increase
Decrease Decrease
Not change Not change
